# A Case of Cephalic Tetanus in an Elderly Patient with Trismus

**DOI:** 10.1155/2018/1247256

**Published:** 2018-06-26

**Authors:** Marilia Bernardes, Saberio Lo Presti, Kenneth Ratzan

**Affiliations:** Department of Medicine, Mount Sinai Medical Center, Miami Beach, FL, USA

## Abstract

We report a case of a 77-year-old woman who presented to the Emergency Room with a three-day history of oral lesions and jaw tightness. Her physical examination was remarkable for the presence of trismus and white ulcers on the visible portion of the tongue. CT head and neck was unremarkable, and she was discharged with empiric treatment for oral candidiasis. She returned two days later with worsening symptoms and subsequently developed tonic-clonic seizures. MRI of the brain and temporomandibular joints were noncontributory. Psychiatry was consulted, and the patient was prescribed olanzapine and mirtazapine for suspected depression with somatization symptoms. She continued to deteriorate despite therapy and developed right lid ptosis and ophthalmoparesis, which led to a presumptive diagnosis of cephalic tetanus. On the 14th day of illness, tetanus immune globulin, metronidazole, and tetanus toxoid vaccine were administered. Despite treatment, the patient died after 24 days of hospitalization. This case illustrates the importance of early recognition of tetanus since successful treatment depends on timely administration of immune globulin.

## 1. Introduction

The incidence of tetanus has steadily declined over the past decades due to universal vaccination. Although it is a rare disease in the developed world, it can be highly lethal, particularly in the elderly population [1, 2]. Cephalic tetanus is a rare form of the disease that is unfamiliar to many physicians and can be easily overlooked or mistaken for other medical conditions. Adverse outcomes may result from delayed recognition of the disease. Herein, we report a delayed diagnosis of tetanus in an elderly female who presented with localized cephalic disease complicated by autonomic dysfunction and aspiration pneumonia, which ultimately led to her death.

## 2. Case Report

A 77-year-old woman presented to the Emergency Room with a three-day history of oral lesions and jaw tightness. She had presented to a different hospital four days prior due to nausea, vomiting, and dysphagia which were deemed secondary to gastroparesis. She received symptomatic treatment and, once stabilized, was discharged home. However, over the next three days, she developed oral sores and jaw tightness, which prompted her to seek medical attention again. Her physical examination revealed normal vital signs, mild dehydration, trismus, and white ulcers on the visible portion of the tongue that were suspicious for oral candidiasis. Laboratory data revealed mild leukocytosis (WBC 11.33 × 10³) and moderate hyponatremia (123 mmol/L) thought to be secondary to volume depletion and low solute intake. A CT scan of the head and neck was unremarkable. The temporomandibular joints were intact. The patient was admitted to the hospital and treated with diazepam, cyclobenzaprine, nystatin, and chlorhexidine mouthwash and was given nutritional supplementation. She was discharged after two days with a plan to follow up as an outpatient. However, she returned two days later with worsening jaw tightness and inability to swallow, for which she was readmitted.

Her past medical history included hypertension, diabetes mellitus, dyslipidemia, and a history of deep venous thrombosis. Prior to admission, medications included glipizide, atorvastatin, telmisartan, and rivaroxaban. She was a lifetime nonsmoker and denied alcohol use. She denied recent trauma or skin lacerations, and reported having completed her primary immunization series against tetanus as a child. Her most recent tetanus booster was in 1965.

On physical exam, blood pressure was 147/82, pulse was 75, respiratory rate was 16, and temperature was 97.9°F (36.6°C). She was euvolemic and in no apparent distress. The presence of trismus was evident—the patient was unable to open her mouth beyond 2 mm. Laboratory data were remarkable for hyponatremia (122 mmol/L), and the patient was placed on normal saline.

The following day, the patient developed three brief episodes of tonic-clonic seizures in the setting of hyponatremia (127 mmol/L) and was treated with intravenous (IV) lorazepam, IV levetiracetam, and 3% saline infusion. An urgent computed tomography (CT) did not reveal any acute intracranial process. The seizures resolved, and the sodium level normalized, but the patient persisted with trismus and dysphagia. As part of the workup, she underwent magnetic resonance imaging (MRI) of the brain and temporomandibular joints, as well as a laryngoscopy and triple-phase bone scan, all of which were noncontributory. She was then given an atypical antipsychotic and antidepressant for a presumptive diagnosis of depression with somatization symptoms. A lumbar puncture was performed, and cerebrospinal fluid analysis revealed 22 nucleated cells (90% lymphocytes), a protein level of 56 mg/dL, and a glucose level of 84 mg/dL. There was no bacterial growth. On the 14th day of illness, the patient developed right lid ptosis and ophthalmoparesis with a right gaze preference, which led to a working diagnosis of cephalic tetanus. She was given intramuscular (IM) tetanus immune globulin, IV metronidazole, and a booster dose of tetanus and diphtheria toxoid vaccine. Baclofen and lorazepam were used for spasm control. Pre-existing anti-tetanus toxoid antibodies were quantified below 0.05 IU/mL.

Unfortunately, despite treatment, she developed episodes of agonal breathing, psychomotor agitation, and autonomic dysregulation characterized by bradycardia-tachycardia with labile blood pressure, which indicated possible progression to generalized tetanus. She had a cardiopulmonary arrest followed by myoclonus status epilepticus complicated with aspiration pneumonia and sepsis, which ultimately led to her death after 24 days of hospitalization.

Postmortem exam of the brain showed moderate to severe hypoxic-ischemic changes. The cerebrum and basal ganglia exhibited vascular congestion, cytoplasmic eosinophilia, and nuclear pyknosis of neuronal cells. Extensive neuronal loss was observed in the hippocampus and cerebellum. The midbrain, pons, and medulla oblongata showed vascular congestion. Pulmonary exam revealed bilateral congestion and focal acute bronchopneumonia.

## 3. Discussion

Tetanus is a life-threatening disease caused by *Clostridium tetani*, a ubiquitous Gram-positive rod-shaped spore-forming bacterium that is commonly found in soil [3]. It is a rare disease in the developed world due to near-universal vaccination, with only 233 cases reported in the United States between 2001 and 2008 [1]. For that reason, many physicians are unfamiliar with its presentation, and the disease can be easily overlooked.

Despite widespread availability of tetanus vaccines, it is not uncommon for patients who completed a primary vaccination series during childhood to fail to receive additional 10-year boosters throughout their lifetime, as seen in this case. Inadequate vaccination is thought to be the main reason behind the higher incidence and lethality of tetanus among the elderly (aged 65 years or more), although decreased immunogenicity appears to play a role in a subset of patients [1, 4–6]. As low as 30% of persons 70 years of age or older have protective antibody levels to tetanus [7]. The patient in the case above was 77 years old and had not received a tetanus booster in over 50 years, which placed her in a category of higher risk for acquiring tetanus.

It is well known that *Clostridium tetani* infects the host by invading the body through breaks in the skin following an injury. Patients with an obvious injury are probably more likely to seek medical care and, therefore, more likely to receive a tetanus booster. However, in up to 25–30% of patients diagnosed with tetanus, a culprit wound is not recognized [8, 9]. This appears to have been the case with our patient. A possible hypothesis is that her mouth ulcers could have served as port of entry for bacteria. This would be consistent with the presentation of the cephalic form of disease, which usually occurs after sustaining an injury to the head or facial area [8, 9]. However, it is unclear whether her ulcers preceded her symptoms or were just a result of trauma in the presence of trismus.

Among the types of clinical presentation of tetanus described in adults, the cephalic form is the least common of them, representing only 1–3% of cases. It classically presents with trismus and cranial nerve involvement. The facial nerve is most commonly affected, although involvement of cranial nerves III, IV, VI, and XII have also been described [10, 11]. Cephalic tetanus frequently progresses to generalized tetanus and is associated with a high mortality [8, 9, 11, 12]. Our patient presented with the classic findings of cephalic tetanus, including trismus, dysphagia, and cranial nerve involvement. She later progressed to generalized tetanus, as indicated by development of agonal breathing, psychomotor agitation, and autonomic dysregulation.

The diagnosis of tetanus is based on clinical findings and epidemiological information, since there is no confirmatory laboratory test. An undetectable serologic result prior to administration of immunoglobulins may provide additional support for diagnosis, although the disease has been described in the presence of “protective” antibody levels [2]. Furthermore, tetanus can be mistaken for many other medical conditions, including drug-induced dystonic reactions, meningitis, seizures, hypocalcemia, rabies, strychnine poisoning, and stroke, but most lack the characteristic features of the disease [9]. In fact, our patient was initially diagnosed with oral candidiasis, and the presence of trismus was thought to be secondary to a local infection. She subsequently developed seizures, which were thought to be secondary to symptomatic hyponatremia. However, a sodium level of 127 mmol/L would not be expected to cause seizures, especially in the setting of chronic hyponatremia. Even at levels below 120 mmol/L, seizures occur in only 1 to 5% of cases of severe hyponatremia [13, 14]. On the other hand, tetanic spasms, a classic manifestation of tetanus, can be difficult to distinguish from seizures [15], and perhaps could have been what our patient experienced. Her symptoms persisted despite initial therapy, and she later received a diagnosis of depression with somatization symptoms. However, none of the abovementioned diagnoses fully explained her symptoms.

It was only on the 14th day of illness that our patient was finally treated for tetanus, but her clinical picture continued to deteriorate. The failure to respond to therapy is most likely explained by the inability of immune globulins to neutralize toxins that are already bound to nerve endings, underlying the importance of early recognition, and prompt initiation of treatment. Although current evidence suggests a better outcome with intrathecal immune globulin administration, compared to the intramuscular route, it is unclear whether this approach is superior in the subset of patient with cephalic tetanus [12, 16].

Previous autopsy studies done in patients who died from tetanus have failed to reveal a pathognomonic finding. Most findings are nonspecific and include various degrees of cerebral edema, congestion, and degenerative changes. Bronchopneumonia is also a very common finding [17]. The postmortem exam in our patient was consistent with these findings.

## 4. Conclusion

The patient in the case depicted above was an elderly female who was susceptible to tetanus due to inadequate vaccination, as evidenced by her undetectable anti-tetanus antibody levels. She presented with typical symptoms of an unfamiliar disease in the developed world, which is often overlooked or misdiagnosed. The lack of diagnostic laboratory tests further contributed to a delay in the diagnosis of tetanus and despite treatment, her condition continued to deteriorate. This case illustrates the importance of early recognition of tetanus, including the localized and cephalic forms of disease, since successful treatment depends on timely administration of immune globulin. Furthermore, this case reminds us of the importance of adequate immunization as the most effective intervention against tetanus.
